# Morphological and Morphometric Analysis of the Pterion and Its Neurosurgical Implications

**DOI:** 10.7759/cureus.104757

**Published:** 2026-03-06

**Authors:** Aruna Arya, Sushma Tomar, Nikhil Aggarwal, Punita Manik

**Affiliations:** 1 Anatomy, Muzaffarnagar Medical College, Muzaffarnagar, IND; 2 Anatomy, King George’s Medical University, Lucknow, IND; 3 Anatomy, Army College of Medical Sciences, New Delhi, IND

**Keywords:** craniometry, neurosurgery, pterion, skull, zygomatic arch

## Abstract

Background

The pterion is an important craniometrical landmark located on the lateral aspect of the skull, formed by the articulation of the frontal, parietal, squamous temporal, and greater wing of the sphenoid bones. Owing to its close relationship with vital neurovascular structures, such as the anterior division of the middle meningeal artery and the Sylvian fissure, precise knowledge of its morphology and location is essential for neurosurgical interventions, anthropological assessment, and forensic evaluation. This study aimed to investigate the morphological types of pterion and to determine its morphometric relationship with selected bony landmarks in human skulls.

Methodology

This cross-sectional study was conducted on 100 adult human dry skulls (200 sides) of unknown age and sex, obtained from the Departments of Anatomy at King George’s Medical University, Lucknow, and Muzaffarnagar Medical College, Muzaffarnagar, Uttar Pradesh, India, from December 2022 to November 2023. Pterion was classified into four types based on the sutural pattern. The center of the pterion was identified, and linear distances were measured from this point to the superior border of the zygomatic arch (P-ZA) and the posterolateral margin of the frontozygomatic suture (P-FZS) using a digital vernier caliper with a precision of 0.01 mm. Observations were recorded bilaterally and subjected to statistical analysis using SPSS version 26 (IBM Corp., Armonk, NY, USA).

Results

Of the 200 pterions examined, the sphenoparietal type was the most common, observed in 160 (80%) sides, followed by the stellate type in 21 (10.5%) sides, the epipteric type in 11 (5.5%) sides, and the frontotemporal type in eight (4%) sides. On the right side, the sphenoparietal pterion was present in 76 (38%) sides, while on the left side, it was seen in 84 (42%) sides. The mean distance from the centre of pterion to the frontozygomatic suture (P-FZS) was 32.82 ± 4.66 mm on the right side and 32.16 ± 4.40 mm on the left side. The mean distance from the centre of pterion to the superior border of the zygomatic arch (P-ZA) was 37.51 ± 4.16 mm on the right side and 36.50 ± 4.13 mm on the left side.

Conclusions

The present study highlights considerable morphological and morphometric variability of the pterion. A thorough understanding of these variations is crucial for neurosurgeons to accurately localize burr holes and minimize surgical complications. The findings also contribute valuable reference data for anthropologists and forensic experts.

## Introduction

The primary objective of modern neurosurgical practice is to achieve maximal therapeutic benefit with minimal invasiveness. This necessitates a precise understanding of cranial bony landmarks, sutures, and foramina, which serve as reliable guides during surgical approaches to intracranial pathologies. Among these landmarks, the pterion is one of the most frequently utilized external reference points in neurosurgery due to its consistent anatomical location and its close association with vital neurovascular structures.

The pterion is located on the norma lateralis of the skull, at the floor of the temporal fossa, deep to the temporalis muscle. It represents the region where the frontal bone, parietal bone, squamous part of the temporal bone, and the greater wing of the sphenoid bone converge to form an H-shaped sutural pattern [1]. Conventionally, the pterion is described as lying approximately 4 cm superior to the zygomatic arch and 3-3.5 cm posterior to the frontozygomatic suture, making it clinically accessible even when advanced neuronavigation tools are unavailable [2,3].

Deep to the pterion lie several critical anatomical structures, including the anterior division of the middle meningeal artery, middle meningeal vein, stem of the lateral (Sylvian) sulcus, insula, middle cerebral vessels, and, on the dominant hemisphere, Broca’s motor speech area [4-6]. Consequently, trauma to the pterional region may result in rupture of the middle meningeal artery, leading to life-threatening extradural hematoma, which mandates prompt surgical intervention [7]. For this reason, the pterion serves as a key landmark for burr hole placement and pterional craniotomy during evacuation of epidural hematomas and for surgical access to lesions involving the anterior and middle cranial fossae [8,9].

In the neonatal skull, the pterion corresponds to the anterolateral (sphenoidal) fontanelle, which closes within the first three to four months after birth. This fontanelle plays a vital role during childbirth by permitting moulding of the fetal head and facilitating rapid postnatal brain growth [10]. Beyond its neurosurgical relevance, the pterion has also gained importance in anthropology and forensic science, where its sutural morphology has been utilized for age estimation, sex determination, and population-based cranial studies [11,12].

Morphologically, the pterion exhibits distinct sutural patterns, which have been classically categorized based on the manner of articulation of the participating cranial bones. Broca initially described three types of pterion, which were later expanded by Murphy to include a fourth variant [10,11]. The sphenoparietal type is the most common configuration, in which the greater wing of the sphenoid articulates directly with the parietal bone, separating the frontal and temporal bones from direct contact. In the frontotemporal type, the frontal bone comes into direct contact with the squamous part of the temporal bone, preventing articulation between the sphenoid and parietal bones. The stellate type is characterized by the convergence of the frontal, parietal, temporal, and sphenoid bones at a single point, producing a star-shaped or radiating sutural pattern. The epipteric type is distinguished by the presence of one or more small sutural (wormian) bones interposed between the parietal bone and the greater wing of the sphenoid, which may alter the apparent location of the pterion and pose challenges during radiological interpretation and surgical procedures [13,14]. Sutural variations of the pterion are influenced by genetic determinants governing ossification timing, mechanical forces exerted by temporalis muscle traction, and population-specific cranial growth patterns [11,15-17]. Environmental influences such as nutrition and biomechanical stress during cranial development have also been proposed as contributory factors [15,16]. These factors may collectively account for ethnic differences observed in sutural morphology.

Among these variants, the epipteric type holds particular clinical significance. During neurosurgical procedures, drilling over an epipteric bone may lead to unintentional penetration into the orbital cavity, increasing the risk of iatrogenic injury [18]. Furthermore, operative approaches to the optic canal, sphenoid ridge, and anterior circulation aneurysms frequently utilize the pterional corridor, underscoring the necessity for precise localization of the pterion in relation to palpable bony landmarks [19].

Given the clinical, anthropological, and forensic importance of the pterion, a detailed morphological and morphometric analysis is essential. The present study was therefore undertaken to evaluate the types of pterion and their morphometric relationship with the zygomatic arch and frontozygomatic suture in human skulls, with the aim of providing region-specific anatomical data that may assist neurosurgeons in safer surgical planning and execution.

## Materials and methods

The present study was conducted as a cross-sectional observational study in the Departments of Anatomy at King George’s Medical University, Lucknow, and Muzaffarnagar Medical College, Muzaffarnagar, Uttar Pradesh, India, during the period from December 2022 to November 2023. A total of 100 adult human dry skulls of unknown age, sex, and origin were examined. The skulls were procured from the anatomical museums of the respective institutions. As observations were made bilaterally, a total of 200 pterions, comprising 100 right-sided and 100 left-sided pterions, were included in the study.

Only skulls with normal morphology, without gross deformity, erosion, or defacement, and with the skull cap removed superior to the level of the pterion were included to ensure clear visualization of sutural patterns and accurate measurements. Skulls showing bilateral fractures of the zygomatic bone or any damage involving the pterional region or adjacent bony landmarks were excluded, as such defects could interfere with precise morphometric assessment.

The sample size comprised all available adult dry skulls that satisfied the inclusion criteria during the study period and thus represented a convenience sample. As the study was descriptive and based on existing osteological collections, no prior formal sample size calculation was performed. This has been acknowledged as a methodological limitation. However, the total sample of 200 pterions is comparable to or exceeds that of several previously published morphometric studies of the pterion [6-9].

The pterion was identified on the lateral aspect of each skull at the junction of the frontal, parietal, squamous temporal, and greater wing of the sphenoid bones. Based on the sutural configuration and pattern of articulation of these bones, the pterion was classified into one of the following four types: sphenoparietal, frontotemporal, stellate, or epipteric, according to Murphy’s classification [11]. Particular attention was paid to the identification of sutural (wormian) bones in cases of epipteric pterion.

**Figure 1:**
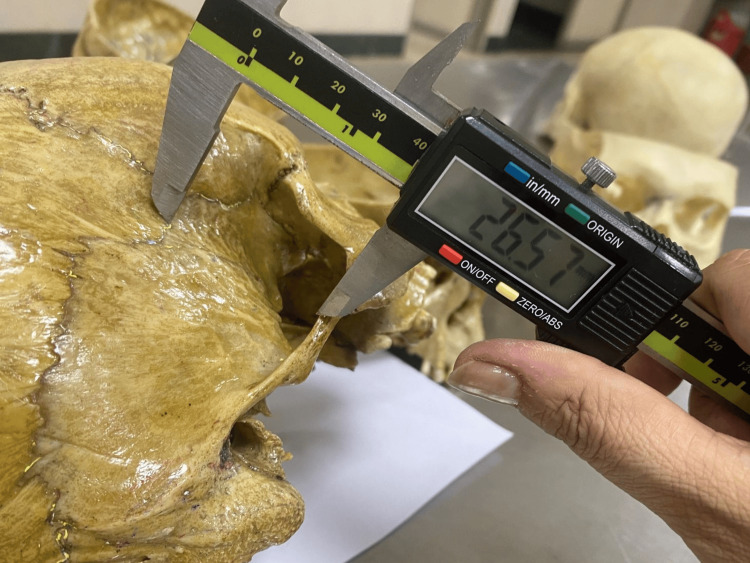
Measurement of the linear distance from the centre of the pterion to the superior border of the zygomatic arch (P–ZA) using a digital vernier caliper.

**Figure 2:**
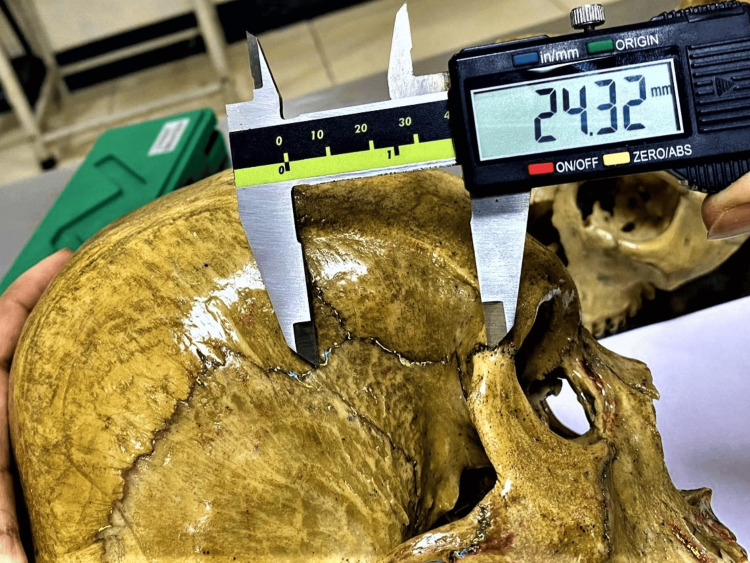
Measurement of the linear distance from the centre of the pterion to the posterolateral margin of the frontozygomatic suture (P–FZS) using a digital vernier caliper.

The center of the pterion was identified as the point of maximum sutural convergence, defined as the anatomical locus where the frontal, parietal, squamous temporal, and greater wing of the sphenoid bones meet most centrally, corresponding to the classical description of the pterion in standard osteological and morphometric literature [1,3,11]. This point was visually confirmed in accordance with previously published morphometric studies [6-9] and is illustrated in Figure 1 and Figure 2. This point was used as the reference landmark for all linear measurements, in accordance with the methodology adopted in several published morphometric studies on the pterion.

From the identified center of the pterion, two linear distances were measured on each side of the skull: the distance to the superior border of the zygomatic arch (P-ZA) and the distance to the posterolateral margin of the frontozygomatic suture (P-FZS). These measurements were obtained using a digital vernier caliper with a precision of 0.01 mm, and the measurement techniques are illustrated in Figure 1 and Figure 2.

The measurement to the zygomatic arch (P-ZA) was taken to the midpoint of the superior border of the arch, directly inferior to the pterion. The midpoint was determined by identifying the anterior and posterior extremities of the zygomatic arch and calculating the central point along its superior margin using direct linear assessment with a digital caliper. This ensured reproducibility and consistency across specimens.

All measurements were performed using a calibrated digital vernier caliper (precision 0.01 mm) following a uniform protocol. Each parameter was measured twice by the same trained observer at two separate sittings spaced one week apart. In cases where the difference between the two readings exceeded 0.5 mm, a third measurement was obtained, and the median value was recorded for analysis. If the difference was ≤0.5 mm, the mean of the two readings was calculated and documented. This approach minimized intraobserver variability and enhanced measurement reliability. Further, these measurements were taken with the caliper held perpendicular to the bony surface to minimize parallax and instrumental error. The measurements were recorded systematically for both right and left sides. Morphological data were expressed as frequencies and percentages, while morphometric data were analysed via SPSS version 26 (IBM Corp., Armonk, NY, USA) using descriptive statistics, including minimum values, maximum values, mean, and standard deviation.

## Results

The present study analyzed a total of 100 adult human dry skulls, yielding 200 pterions (100 right-sided and 100 left-sided) for evaluation of morphological types and morphometric parameters.

Among the 200 pterions examined, the sphenoparietal type was the most prevalent, accounting for 160 (80%) pterions. This was followed by the stellate type, observed in 21 (10.5%) pterions, the epipteric type in 11 (5.5%) pterions, and the frontotemporal type, which was the least common, seen in eight (4%) pterions. On side-wise analysis, sphenoparietal pterion was observed in 76 (38%) right-sided pterions and 84 (42%) left-sided pterions. The stellate type was noted in 12 (6%) right sides and nine (4.5%) left sides, while the epipteric type was present in six (3%) right sides and five (2.5%) left sides. The frontotemporal type showed the lowest frequency, occurring in six (3%) right sides and two (1%) left sides. Overall, the distribution demonstrated a clear predominance of the sphenoparietal configuration on both sides of the skull. Table 1 highlights that the sphenoparietal type constituted the dominant morphological pattern of pterion in the studied sample, whereas frontotemporal pterion was relatively uncommon.

**Table 1:** Frequency distribution of different morphological types of pterion on the right and left sides of the skull (n = 200 pterions from 100 skulls).

Pterion type	Right side n (%)	Left side n (%)	Total n (%)
Sphenoparietal	76 (38%)	84 (42%)	160 (80%)
Frontotemporal	6 (3%)	2 (1%)	8 (4%)
Stellate	12 (6%)	9 (4.5%)	21 (10.5%)
Epipteric	6 (3%)	5 (2.5%)	11 (5.5%)
Total	100 (50%)	100 (50%)	200 (100%)

**Figure 3:**
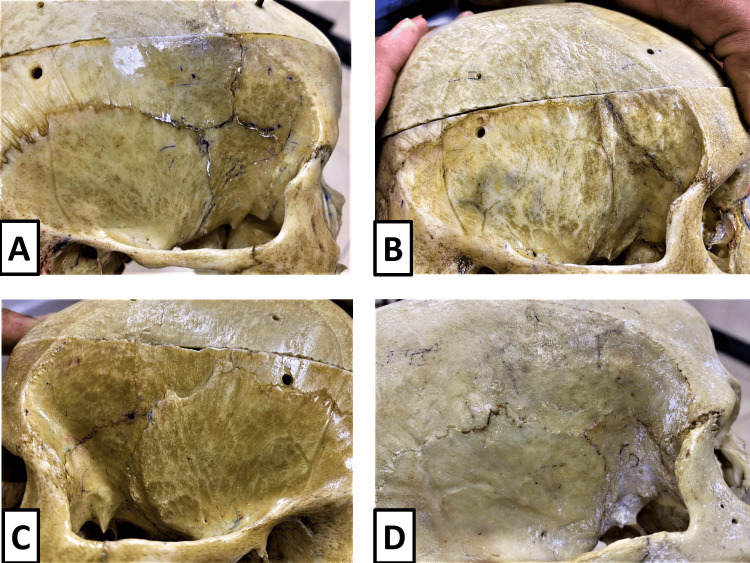
Morphological variants of pterion observed in the present study. (A) Sphenoparietal type, (B) frontotemporal type, (C) stellate type, and (D) epipteric type showing the presence of a sutural (wormian) bone.

The four morphological variants of pterion identified in the present study, namely, sphenoparietal, frontotemporal, stellate, and epipteric, are illustrated in Figure 3. The figure demonstrates the distinct sutural patterns formed by the participating cranial bones, with epipteric pterion showing the presence of sutural (wormian) bone interposed between the parietal bone and the greater wing of the sphenoid.

The descriptive statistics of the linear measurements from the center of the pterion to selected bony landmarks are presented in Table 2. The distance from the center of the pterion to the posterolateral margin of the frontozygomatic suture (P-FZS) on the right side ranged from 21.19 mm to 46.77 mm, with a mean of 32.82 ± 4.66 mm. On the left side, the P-FZS distance varied from 20.72 mm to 40.49 mm, with a mean of 32.16 ± 4.40 mm. The distance from the center of the pterion to the superior border of the zygomatic arch (P-ZA) on the right side ranged between 25.57 mm and 46.73 mm, with a mean of 37.51 ± 4.16 mm. On the left side, this distance ranged from 24.92 mm to 45.51 mm, with a mean of 36.50 ± 4.13 mm. Overall, the morphometric data demonstrate that the pterion is consistently located approximately 3.2-3.3 cm posterior to the frontozygomatic suture and 3.6-3.8 cm superior to the zygomatic arch, with minor side-to-side variations.

**Table 2:** Descriptive statistics of linear measurements from the center of the pterion to the frontozygomatic suture (P-FZS) and superior border of the zygomatic arch (P-ZA) on the right and left sides of the skull (values expressed in millimeters; n = 100 each side).

Measured parameters	N	Minimum	Maximum	Mean	Standard deviation
Right P-FZS (in mm)	100	21.19	46.77	32.8243	4.65796
Left P-FZS (in mm)	100	20.72	40.49	32.1613	4.39929
Right P-ZA (in mm)	100	25.57	46.73	37.5093	4.15723
Left P-ZA (in mm)	100	24.92	45.51	36.5027	4.13109

Thus, the present study demonstrates that the sphenoparietal type is the most prevalent morphological variant of the pterion, while the frontotemporal type is the least common. The morphometric analysis shows that the pterion maintains a relatively consistent anatomical relationship with the frontozygomatic suture and the superior border of the zygomatic arch on both sides, with only minimal side-to-side variation. These observations establish baseline morphological and morphometric data for the pterion in the studied sample.

## Discussion

The pterion serves as a critical anatomical and surgical landmark owing to its close relationship with the anterior division of the middle meningeal artery, Sylvian fissure, and underlying cerebral cortex. From a neurosurgical standpoint, precise localization of the pterion is crucial during emergency burr hole placement for extradural hematoma, particularly when neuronavigation is unavailable. Knowledge of the average distance of the pterion from the frontozygomatic suture and the zygomatic arch enables rapid surface marking in trauma settings. In cases of anterior circulation aneurysms, sphenoid ridge meningiomas, and lesions of the Sylvian fissure, the pterional craniotomy provides optimal access with minimal cortical retraction. Variations such as the epipteric type may alter the expected bony configuration and mislead intraoperative drilling, thereby increasing the risk of orbital penetration or incomplete decompression [6-9,18,19]. Therefore, preoperative awareness of sutural morphology is not merely descriptive but directly influences surgical planning, bone flap design, and operative safety.

In the present study, the sphenoparietal type of pterion was the most frequently observed variant (80%), followed by the stellate type (10.5%), epipteric type (5.5%), and frontotemporal type (4%). This predominance of the sphenoparietal type is comparable to findings of other studies, which have consistently reported it as the most common pterion configuration [6-8,11,13,14,20]. Studies conducted on Indian populations by Agrawal et al., Prasad and Rout, and Gupta et al. have reported sphenoparietal frequencies ranging from approximately 70% to over 85%, supporting the observations of the present study [7-9]. Similar findings have also been documented in Australian, Nigerian, Turkish, and Japanese populations [11,15,19].

Variations across studies may also reflect methodological differences. Certain authors identified the pterion center as the geometric midpoint of sutural intersections [6,9], whereas others used visual estimation of sutural convergence [7,8]. Minor inconsistencies in caliper positioning or landmark identification can produce measurable differences in morphometric values. Such methodological variability underscores the importance of clearly defining measurement protocols when comparing morphometric data across populations.

The evolutionary explanation proposed by Murphy and later authors provides a plausible basis for this predominance, suggesting that progressive neurocranial expansion in humans leads to incorporation of the anterosuperior part of the temporal bone into the greater wing of the sphenoid, resulting in direct sphenoid-parietal articulation [11,19]. This evolutionary adaptation is believed to accommodate increasing brain volume and is less commonly seen in non-human primates, where the frontotemporal type is more prevalent [17].

The stellate type, observed in 10.5% of pterions in the present study, was the second most common variant. This frequency lies within the broad range reported in Indian studies, where stellate pterion has been documented to vary between approximately 2% and 15% [7,8,13,17]. However, some studies from Turkish and African populations have reported either a very low or a complete absence of the stellate type, highlighting ethnic and regional differences in sutural morphology [15,16]. Such variations may be attributed to differences in genetic makeup, environmental influences, and biomechanical forces acting on cranial sutures during growth and development.

The epipteric type, identified in 5.5% of pterions in the present study, is of particular clinical relevance. Comparable frequencies have been reported in several Indian studies, although higher incidences have been documented in certain populations [7-9,13,18]. The formation of epipteric bones has been linked to the presence of additional ossification centers along the sutural margins, resulting in small, irregular sutural bones interposed between the parietal bone and the greater wing of the sphenoid [15]. From a clinical standpoint, these sutural bones may mimic fracture lines on radiological imaging and may lead to erroneous surgical localization if not recognized preoperatively [8,18]. In neurosurgical practice, drilling over an epipteric bone instead of the true pterional center may result in inadequate decompression or inadvertent orbital penetration.

The frontotemporal type, which was the least common variant in the present study (4%), has similarly been reported as a rare finding in most Indian studies [7,8,14]. Its low prevalence in modern human skulls further supports the phylogenetic theory that this configuration is more characteristic of lower primates and gradually diminishes with human evolution [11,17].

Morphometric analysis in the present study demonstrated that the mean distance from the center of the pterion to the frontozygomatic suture (P-FZS) was 32.82 ± 4.66 mm on the right side and 32.16 ± 4.40 mm on the left side, while the mean distance to the superior border of the zygomatic arch (P-ZA) was 37.51 ± 4.16 mm on the right side and 36.50 ± 4.13 mm on the left side. These values are comparable to those reported by Agrawal et al., Gupta et al., and Muche et al., where the pterion was found to be approximately 3-3.5 cm posterior to the frontozygomatic suture and 3.5-4 cm superior to the zygomatic arch [6-9]. Minor side-to-side variations observed in the present study have also been reported by other authors and are generally considered clinically insignificant [6,7,9].

The relatively consistent morphometric relationship of the pterion with easily identifiable bony landmarks reinforces its utility as a reliable external guide during neurosurgical procedures, particularly in emergency settings where neuronavigation facilities may not be available. The findings of the present study, therefore, add valuable region-specific data to the existing literature and further emphasize the importance of detailed anatomical knowledge of the pterion for neurosurgeons, anatomists, and forensic experts. Although most studies report predominance of the sphenoparietal type, variability in reported frequencies highlights the influence of ethnicity, sampling strategy, and methodological definitions of sutural intersection. Future multicentric studies incorporating radiological correlation and demographic stratification may provide deeper insight into observed discrepancies.

Limitations of the study

The present study was conducted on dry adult human skulls of unknown age, sex, and origin; therefore, correlations with demographic parameters such as sex-specific or age-related variations in pterion morphology could not be assessed. As the study was performed on osteological specimens, the relationship of the pterion with soft tissue structures and underlying neurovascular elements could not be evaluated. Additionally, a formal sample size calculation was not performed before study initiation, as the research was based on available osteological specimens. The use of a convenience sample may limit the generalizability of the findings. Thus, the findings represent observations from a limited regional sample and may not be universally applicable to all populations. Despite these limitations, the study provides reliable morphological and morphometric data that are relevant for clinical, anatomical, and forensic applications.

## Conclusions

The present study highlights the morphological diversity and consistent morphometric relationships of the pterion in adult human skulls. The sphenoparietal type was identified as the most prevalent variant, while the frontotemporal pterion was the least common. The measured distances of the pterion from the frontozygomatic suture and the superior border of the zygomatic arch showed relatively uniform values with minimal side-to-side variation, reinforcing the reliability of these landmarks for surgical localization. A detailed understanding of these variations is essential for neurosurgeons to accurately plan pterional approaches and minimize the risk of iatrogenic injury. The findings of this study also contribute valuable anatomical data for anthropological and forensic investigations.
